# Young people’s smoking and vaping behaviour, and comparative perceptions of appeal, imagery and harm, across different vape devices and a tobacco cigarette: findings from UK cross-sectional surveys in 2020 and 2023

**DOI:** 10.3389/fpubh.2025.1689766

**Published:** 2025-12-18

**Authors:** Anne Marie MacKintosh, Danielle Mitchell, Shona Hilton, Marissa Smith, Allison Ford

**Affiliations:** 1 Institute for Social Marketing and Health, University of Stirling, Stirling, United Kingdom; 2 Department of Social Work and Social Policy, University of Strathclyde, Glasgow, United Kingdom; 3 School of Health and Wellbeing, University of Glasgow, Glasgow, United Kingdom

**Keywords:** disposable vapes, E-cigarettes, young people, survey, cross-sectional, vaping, smoking, susceptibility

## Abstract

**Introduction:**

After the introduction of a new generation of disposable vapes (e.g., Elf Bar) in the UK in 2021, there was a rapid increase in their use by young people and concern about their availability and marketing. This study examined young people’s vaping and smoking between 2020 and 2023, and perceptions of a disposable vape, a tank model and a traditional cigarette, to examine whether disposable vapes may have contributed to the rise in youth vaping.

**Methods:**

Online cross-sectional surveys in 2020 (Youth Tobacco Policy Survey *n* = 2,121) and 2023 (Youth E-cigarettes Policy Survey *n* = 2,164) with 11–16-year-olds across the UK were conducted. Measures included demographics, vaping and vaping susceptibility, smoking and smoking susceptibility, type(s) of vapes used, and product ratings across 11 items/attributes covering product appeal, imagery and perceptions of harm.

**Results:**

Between 2020 and 2023, prevalence of ever smoking reduced from 12.2 to 9.3%, whilst ever vaping increased from 10.1 to 17.5%. Vaping experimentation was not confined to young people who had already tried smoking: in 2023, never smokers accounted for the majority of ever vapers (56.6%). Of those who had tried vaping, disposable vapes were the most commonly used device type, with 72.8% having used a disposable vape the first time they tried vaping. There was an association between having tried vaping and susceptibility to smoke. Disposable vapes were rated more favourably compared with a tank device on 9 of 11 items across appeal, imagery and harm (range adjusted odds ratio (AOR): 1.40–4.16; *p* < 0.001). Traditional cigarettes were rated less favourably than a tank device on all items (range AOR: 0.08–0.61; *p* < 0.001).

**Conclusion:**

This study highlights the appeal of disposable vapes to young people. Causality cannot be inferred due to the cross-sectional design of the study, however, positive perceptions of disposable vapes across dimensions of appeal, imagery and harm, may have contributed to trends in youth vaping. Further monitoring of the nicotine market, product marketing, and young people’s response is critical, particularly as the market adapts in response to the disposable vapes ban.

## 1 Introduction

After the introduction of a new generation of disposable vapes (e.g., Elf Bar) in the United Kingdom (UK) in 2021, there was a rapid increase in their uptake and use by young people (1, 2), and growing concern about their widespread availability and marketing (3). The proportion of 11–17-year-olds in Great Britain who reported having tried vaping increased from 13.9% in 2020, to 20.5% in 2023, after which the trend appears to have plateaued (1). In 2023, 69% of young people who currently vaped most frequently used disposable devices (1). Similar trends were found in the United States, Australia, and Canada (4–6). Disposable vapes also became increasingly popular amongst young adults in the UK during this time (7).

Although it is illegal to sell vapes containing nicotine to anyone under 18 years or to buy them on behalf of anyone under 18 years (a proxy purchase offence), vapes are easily accessible to young people. A UK-wide survey with 11–17-year-olds conducted in May 2024, found that amongst those who had vaped in the last 12 months, 59% had been given vapes from someone they knew and 54% had bought vapes, including by asking others to buy them for them (8). Young people who purchase vapes themselves in shops, consistently report access via smaller stores such as corner shops, newsagents and off-licences (1, 8). Qualitative research with young people aged 11–17 years in England and Scotland, found that retail marketing practices, particularly brightly coloured, eye-catching shop window displays and posters, commonly a feature in smaller shops, and which advertised disposable products, facilitated accessibility to young people by sending a message that these products were targeted at them (8). In the same study, young people who vaped reported that it was easy to know which retailers would sell to people underage and this knowledge was shared amongst peers (8).

In addition to perceived ease of access (8), a combination of product attributes likely account for the appeal of disposable vapes. ‘better flavour/taste’, ‘less expensive’, ‘easier to get’, and ‘smoother to inhale’, were the main reasons for choosing disposable vape brands in a survey of 16–29-year-olds in England (9). Qualitative research with 16–20-year-olds who regularly vaped, similarly highlighted the importance of disposable product attributes such as low price, attractive designs, colours, names and flavours (10). A qualitative study with a younger sample of 11–16-year-olds in Scotland found positive imagery surrounding disposables, which were termed ‘cool’ and ‘fashionable’ (11). These young participants believed their age group were being targeted by vape companies with brightly coloured devices and sweet flavours, and that other types of vaping devices, such as tank models, were for older adults (11).

To tackle the rise in youth vaping and the environmental impact of single use products, on 1st June 2025, all four UK governments (England, Scotland, Wales and Northern Ireland) introduced a ban on the sale and supply of disposable vapes using powers under the Environmental Protection Act 1990 (12). Prior to the ban, an estimated 5 million disposable vapes were thrown away per week, raising concerns around the lack of recycling of useful materials present in batteries, and fire risk (13). Early examination of sales data suggests that disposable vapes continue to be sold post-ban in some shops (14). To further reduce the appeal and availability of vapes to young people, the Tobacco and Vapes Bill is currently progressing through the UK Parliament, giving the government power to restrict the flavours, packaging, retail displays, and advertising of all types of vaping products, and to future-proof against any new types of devices which may emerge (15).

Since their emergence on the market, there has been limited research into young people’s perceptions of disposable devices relative to other types of vaping devices and traditional cigarettes. Refillable devices are more closely linked with smoking cessation. For example, smoking cessation trials focus on refillable devices (to date, there are no published smoking cessation trials with disposable vapes) (16), and reported youth use is lower than for disposable vapes (1, 8). In this paper, we aim to explore what may have contributed to the rise in popularity of disposable vapes compared with refillable devices and traditional cigarettes. Whilst ever smoking amongst 11–17-year-olds in the UK has been in constant decline, there was a recent significant increase from 14% in 2023 to 21% in 2025 of 11–17-year-olds reporting having tried smoking (1). In this paper we present findings from two cross-sectional surveys with 11–16-year-olds in the UK, to explore (1) young people’s smoking, vaping, and susceptibility to smoking and vaping in 2020 and 2023, (2) type of vape used in 2023, and (3) comparative ratings of a disposable vape, a tank model and a traditional cigarette across 11 items exploring key attributes of appeal, product imagery, and perceptions of harm.

## 2 Materials and methods

### 2.1 Design and recruitment

Data were collected in two cross-sectional online surveys in December 2020 and July 2023, as part of the Youth Tobacco Policy Survey (YTPS) and Youth E-cigarette Policy Survey (YEPS) respectively. YEPS is an extension of the long-running repeat cross-sectional YTPS which examined young people’s responses to tobacco control policies in the UK (17). Whilst the focus of the YTPS was mainly on tobacco, the 2020 wave incorporated several measures on vaping. YEPS is a new survey, using the same methodology as YTPS 2020 but the focus is mainly on vaping whilst retaining some key measures on smoking. Both surveys were conducted with 11–16-year-olds in the UK (*n* = 2,121 in 2020; *n* = 2,164 in 2023), and hosted by YouGov, a market research company, that recruited a non-probability sample, intended to be representative of young people in the UK, from their existing UK panel. Participants under 16 years were approached, by email, through existing adult panel members (e.g., their parents). Those aged 16 were approached directly via email. Age and membership/parental membership of the YouGov UK panel were the only inclusion criteria. Ethical approval was received from the University of Stirling General University Ethics Panel (GUEP 2021 1017/2022 7901 7143).

Sample size was determined based on enabling within-survey subgroup analyses and between-year analyses. The surveys aim for a minimum sample of 2000 per year with a sampling error in the region of ±2.2%. Within each year the sample provides a minimum subgroup sample of 100 per age, within sex, to allow subgroup analyses. Details of the numbers who clicked the survey link, who dropped out or were removed after quality control cheques in 2020 and 2023 are given in Supplementary Table S1.

### 2.2 Measures

Prior to each survey, development work involved public involvement (PI) discussion groups with 11–16-year-olds to develop survey items and inform product choices for the survey images. Follow-up cognitive testing ensured comprehension and suitability of measures. This process was important in ensuring use of the correct terminology and informed the need for, and content of, visual stimuli.

#### 2.2.1 Demographics

Age, sex, social grade and a measure of multiple deprivation (Index of Multiple Deprivation, a quantitative measure based on a participant’s postcode and accounting for a variety of socio-demographic factors) were obtained from information held about respondents by YouGov or survey questions. Social grade was determined by the occupation of the chief income earner in the household (ABC1 = middle class, C2DE = working class).

#### 2.2.2 Smoking and smoking susceptibility

Never smokers were categorised as those who had ‘never tried smoking, not even a puff or two’. Susceptibility, defined by the absence of a firm decision not to smoke, was assessed across three items (18). Never smokers were classed as non-susceptible if they answered, ‘definitely not’ to the questions: ‘If one of your friends offered you a cigarette, would you smoke it?’ and ‘Do you think you will smoke a cigarette at any time during the next year?’ and to the likelihood that ‘you will be smoking cigarettes when 18 years old’. Regular smokers were those who indicated smoking at least one cigarette a week.

#### 2.2.3 Vaping and vaping susceptibility

**Figure 1:**
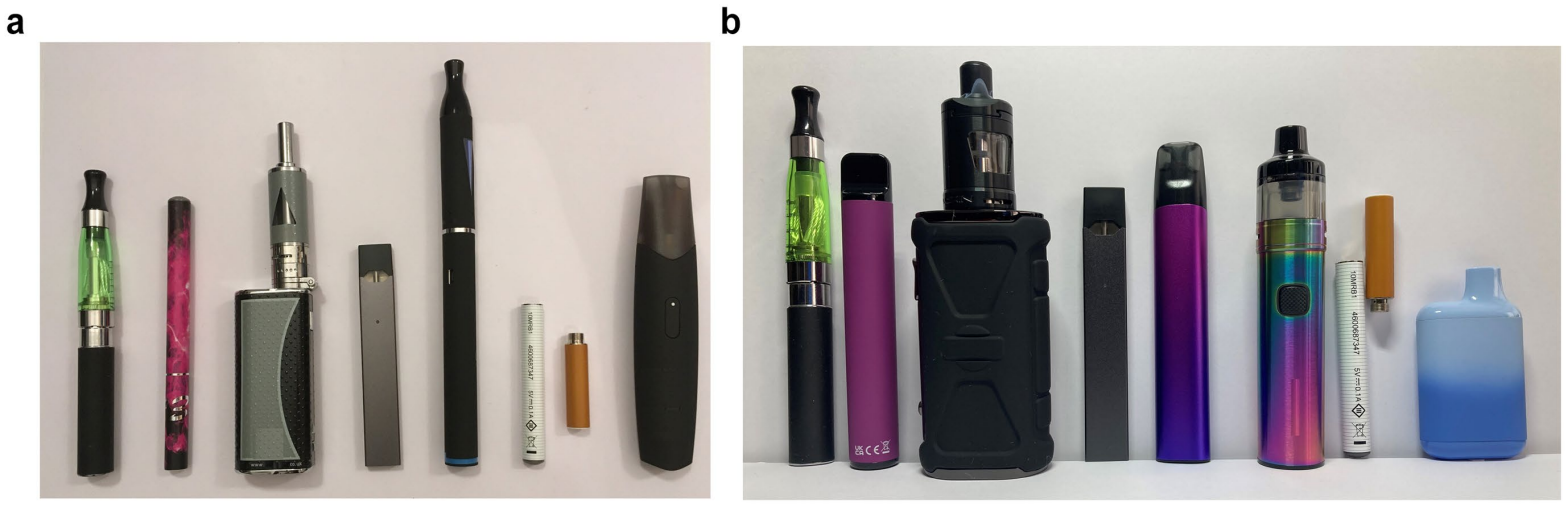
**(a)** Image of vape styles 2020. **(b)** Image of vape styles 2023.

Participants were shown images of vapes to reflect the market in 2020 and 2023 (Figure 1). Never vapers were categorised as those who had ‘never used vapes’ and ever vapers were those who indicated having ‘only ever tried vapes once or twice’; ‘used vapes in the past but never use them now’; ‘occasionally use vapes (less than once a month)’; ‘use vapes at least once a month’ or ‘use vapes at least once a week’. Susceptibility, defined by the absence of a firm decision not to vape, was assessed across three items, adapted from established items of susceptibility to smoking (18–20). Never vapers were classed as non-susceptible if they answered, ‘definitely not’ to the questions: ‘If one of your friends offered you a vape, would you use it?’ and ‘Do you think you will try or use a vape at any time during the next year?’ and to the likelihood that ‘you will be using vapes when 18 years old’. Regular vapers were those who indicated using vapes at least once a week.

#### 2.2.4 Type of vape (2023 only) used

**Figure 2:**
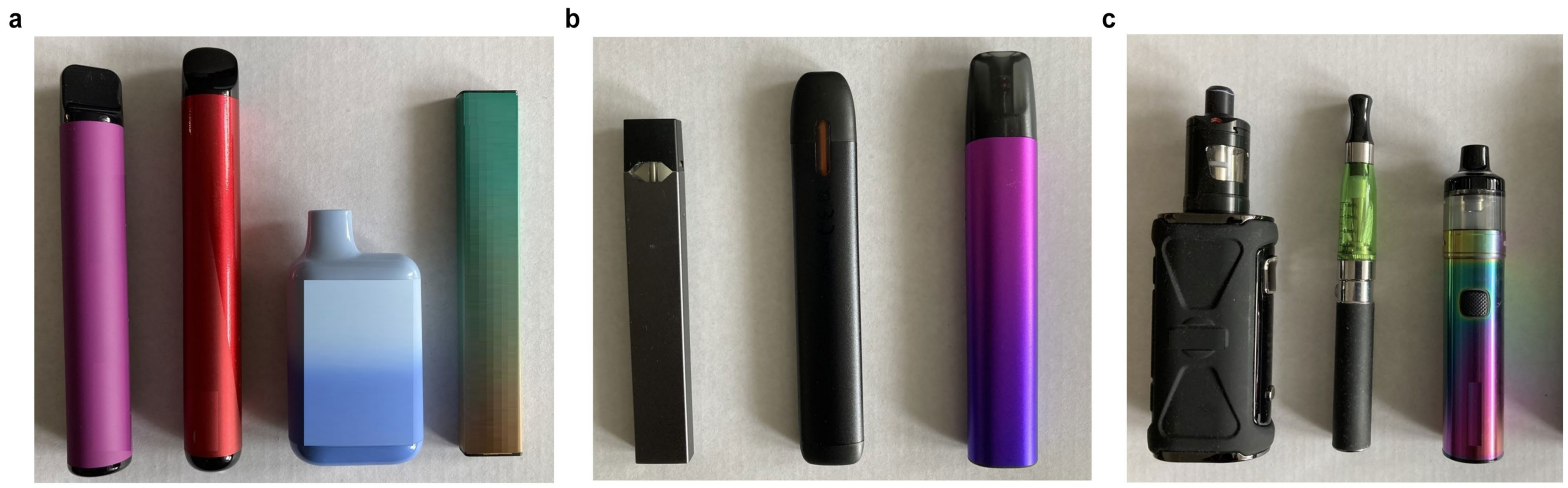
**(a)** Images of different categories of vapes_Disposable. **(b)** Images of different categories of vapes_Rechargeable Pod. **(c)** Images of different categories of vapes_Tank.

Given that the types of vapes available on the market had expanded by 2023, participants were asked about the type of vape they used when they first tried vaping and the type they had used in the past 4 weeks. These were assessed by asking: ‘Thinking about when you FIRST tried vapes/vaping, which of the following best describes the type of vape that you FIRST used/tried?’ and ‘Thinking about the vapes you have used in the PAST 4 WEEKS, which of the following best describes the type(s) you have used in the PAST 4 WEEKS?’. Response categories included text descriptors (A disposable vape (non-rechargeable); Rechargeable with replaceable pre-filled cartridges/pods; and Rechargeable with tank that gets filled with e-liquid) accompanied by images (Figures 2a–c); and an option of ‘Not sure’. A single response was recorded for the type of vape first used whilst multiple responses could be given for the type(s) of vapes used in the past 4 weeks.

#### 2.2.5 Device/product ratings (2023 only)

**Figure 3:**
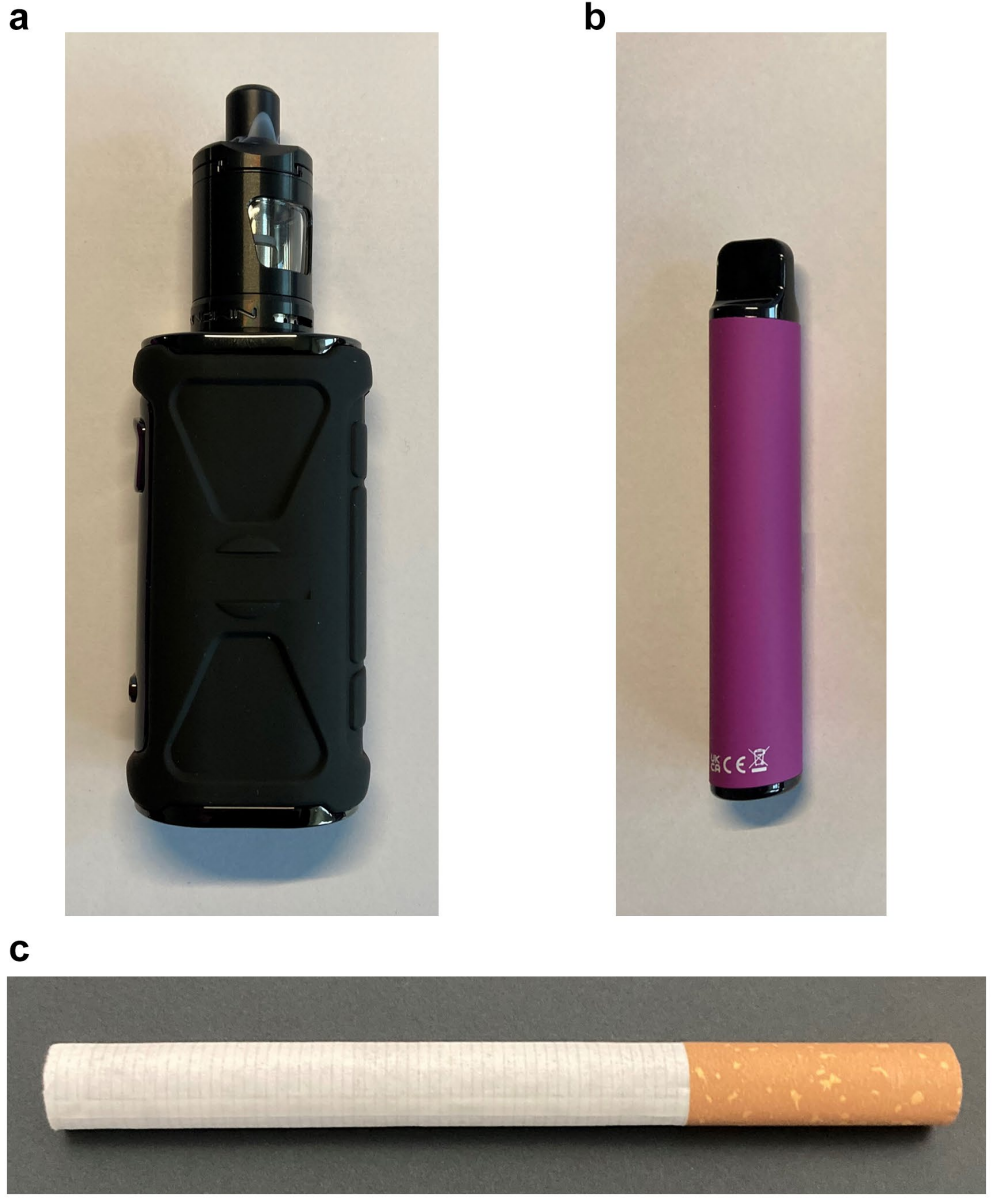
**(a)** Image of tank. **(b)** Image of disposable. **(c)** Image of cigarette.

Participants were shown images of a tank style vape (Figure 3a) a disposable vape (Figure 3b) and a cigarette (Figure 3c). Eleven items assessed young people’s views about tank style vapes, disposable vapes and traditional cigarettes. Participants were asked to rate each on a five-point scale. Four items assessed product appeal: (a) Would not appeal to people my age (1)/would appeal to people my age (5); (b) Unpopular with people my age (1)/popular with people my age (5); (c) Would not appeal to people who have never smoked (1)/would appeal to people who have never smoked (5); (d) I would not be tempted to use this (1)/I would be tempted to use this (5). Three items assessed product imagery: (e) Not cool (1)/cool (5); (f) Boring (1)/fun (5); (g) Looks unpleasant to use (1)/looks pleasant to use (5). Four items related to perceptions of harm: (h) Very harmful to health (1)/Not at all harmful to health (5); (i) Very harmful to the environment (1)/not at all harmful to the environment (5); (j) Very addictive (1); not at all addictive (5); (k) Likely to contain nicotine (1)/unlikely to contain nicotine (5).

Device/product images were shown individually, in a fixed order (Figures 3a–c), with one statement at a time. Each statement was asked of each device/product before moving to the next statement. Half the sample received a version with the above scales reversed so that a favourable rating had a low score (e.g., fun (1)/boring (5)). Participants were also given an option of answering ‘not sure’ for each of the above. For analysis purposes all items were coded such that a low score (1) reflected unfavourable, and a high score (5) reflected favourable perceptions (e.g., boring (1)/fun (5)).

#### 2.2.6 Vaping and/or smoking status

A combined measure of vaping and/or smoking status was derived from the vaping and smoking measures. Participants were classified into one of four categories as follows: (1) those who had ever smoked cigarettes but had never vaped; (2) those who had ever vaped and had also ever smoked cigarettes; (3) those who had ever vaped but had never smoked cigarettes; (4) those who had never smoked cigarettes nor ever vaped.

### 2.3 Selection of products

The PI groups with young people informed which products were selected for the images shown in the survey (Figures 3a–c). Elf Bar was chosen as an easily recognisable disposable device. An example of a tank model provided a comparator to reflect a recognisable ‘earlier generation’ vaping product. An example of a traditional tobacco cigarette was included to explore any differences between smoking and vaping product perceptions. No branding was shown on any device/product.

### 2.4 Analyses

Data were analysed using SPSS v29. In 2020 and 2023, all descriptive statistics were weighted to be representative of the UK population of 11–16-year-olds. Percentages and means presented here are based on weighted data to ensure that the results more closely represent the views of 11–16-year-olds in the UK. The sample weights were provided by YouGov. They used RIM (Random Iterative Method) weighting to match the demographic profile of the sample with the demographic profile of the population in terms of age crossed by gender and region. Chi-square tests were conducted, on weighted data, to test for differences, between 2020 and 2023, in prevalence of vaping, vaping susceptibility, smoking, smoking susceptibility and to test association between smoking susceptibility and ever vaping. To check robustness of the chi-square tests these were repeated on unweighted data and logistic regression was also run on unweighted data, controlling for age, gender and social grade. As all results from the initial chi-square tests were consistent with the sensitivity analyses, details of the sensitivity analyses are not reported in the manuscript.

Paired t-tests were used to produce paired means (weighted) for each of the 11 items for the disposable vs. tank model and cigarette vs. tank model. The tank model was chosen as the reference category as it reflected an earlier generation of vape available prior to the introduction of disposables, originally positioned as a potential aid to smoking cessation. As data from the five-point scale is ordinal, the Wilcoxon signed rank test, was used to test whether ratings differed between the devices/products. Ratings were also dichotomised to produce proportions who gave favourable scores (scores 4–5 being favourable vs. neutral/unfavourable scores 1–3). Bonferroni adjustments were incorporated to account for the multiple comparisons.

As participants rated each of the devices/products across the same 11 items, further analyses needed to account for correlation between individual participants’ ratings of the three devices/products (repeated measures). Generalised estimating equations (GEE) for binary outcomes were used to generate estimates of the likelihood of favourable ratings, comparing the different products/devices, whilst accounting for the repeated measures. In each model the binary outcome was whether participants gave a favourable rating or not (e.g., would appeal to people my age). GEE analyses were run on unweighted data as the analyses controlled for demographic and behavioural variables. An exchangeable correlation structure was used. The GEE analyses also provided a sensitivity analyses for the Wilcoxon signed rank tests on the 11 items. The tank model was again set as the reference category to enable perceptions of a disposable vape to be compared with an earlier generation of vape and for a traditional cigarette to be compared with that same generation of vape. The GEE models controlled for demographics: sex (female vs. male); age group (15–16 vs. 11–12 and 13–14 vs. 11–12); social grade (C2DE vs. ABC1). The GEE also controlled for the combined measure of vaping and smoking status with the reference category set as those who had never smoked nor vaped.

## 3 Results

### 3.1 Sample characteristics

In 2020 and 2023, after weighting, the sample comprised 35% 11–12-year-olds, 33% 13–14-year-olds and 32% 15–16-year-olds (Table 1). Males comprised 51% and the sample was evenly distributed across the quintiles of deprivation.

**Table 1:** Sample characteristics.

	YTPS 2020				YEPS 2023			
	Unweighted		Weighted		Unweighted		Weighted	
	*n*	%	*n*	%	*n*	%	*n*	%
Age group								
11–12	564	26.6%	745	35.1%	760	35.1%	760	35.1%
13–14	738	34.8%	705	33.3%	724	33.5%	719	33.2%
15–16	819	38.6%	671	31.6%	680	31.4%	685	31.6%
Sex								
Male	1,066	50.3%	1,087	51.3%	1,094	50.6%	1,110	51.3%
Female	1,055	49.7%	1,034	48.7%	1,070	49.4%	1,054	48.7%
Social grade								
ABC1	1,435	67.7%	1,422	67.0%	1,614	74.6%	1,594	73.7%
C2DE	686	32.3%	699	33.0%	550	25.4%	570	26.3%
IMD^#^ quintile								
1 – Most deprived	368	17.4%	424	20.0%	320	14.8%	433	20.0%
2	399	18.8%	424	20.0%	399	18.4%	433	20.0%
3	391	18.4%	424	20.0%	441	20.4%	433	20.0%
4	464	21.9%	424	20.0%	469	21.7%	433	20.0%
5 – Least deprived	498	23.5%	424	20.0%	535	24.7%	433	20.0%
Vaping status								
Never vaped	1,887	89.0%	1,906	89.9%	1,806	83.5%	1,786	82.5%
Ever vaped^a^	234	11.0%	215	10.1%	358	16.5%	378	17.5%
Current^b^	55	2.6%	52	2.4%	120	5.5%	127	5.9%
Regular^c^	25	1.2%	22	1.0%	55	2.5%	57	2.6%
Vaping susceptibility^d^								
Susceptible	617	32.7%	629	33.0%	773	42.8%	771	43.2%
Smoking status								
Never smoked	1,846	87.0%	1,863	87.8%	1,980^e^	91.5%	1,961	90.7%
Ever smoked^a^	275	13.0%	258	12.2%	183	8.5%	201	9.3%
Current smoker^b^	70	3.3%	69	3.3%	37	1.7%	41	1.9%
Regular smoker	38	1.8%	37	1.7%	15	0.7%	17	0.8%
Smoking susceptibility^e^								
Susceptible	324	17.6%	336	18.0%	417	21.1%	421	21.5%
Vaping and/or smoking status								
Ever smoked cigarettes but never vaped	116	5.5%	114	5.4%	34	1.6%	37	1.7%
Ever vaped and ever smoked cigarettes	159	7.5%	144	6.8%	149	6.9%	164	7.6%
Ever vaped but never smoked cigarettes	75	3.5%	71	3.3%	209	9.7%	214	9.9%
Never smoked nor vaped	1,771	83.5%	1,792	84.5%	1,771	81.9%	1,748	80.8%
Type of device first used^f^								
Disposable	–	–	–	–	265	74.0%	275	72.8%
Rechargeable (pods)	–	–	–	–	39	10.9%	44	11.7%
Rechargeable (tank)	–	–	–	–	23	6.4%	26	6.8%
Unsure/not specified	–	–	–	–	31	8.7%	33	9.6%
Type of device used past 4 weeks^g^								
Disposable	–	–	–	–	136	78.6%	147	76.5%
Rechargeable (pods)	–	–	–	–	35	20.2%	42	21.7%
Rechargeable (tank)	–	–	–	–	21	12.1%	24	12.4%
Unsure/not specified	–	–	–	–	8	4.6%	10	5.1%

Total sample *n* = 2,121 in 2020, *n* = 2,164 in 2023. #IMD, Index of multiple deprivation. ^a^Including current and regular; ^b^Including regular; ^c^Regular vaping (more than once a week); ^d^Amongst those who have never tried vapes; ^e^Missing cases = 1; ^e^Smoking susceptibility amongst never smokers; ^f^Base = ever vapers 2023 (*n* = 358/378 unweighted/weighted); ^g^Base = vaped in past month 2023 (*n* = 173/192 unweighted/weighted).

#### 3.1.1 Smoking

Prevalence of ever smoking decreased from 12.2% (*n* = 258) in 2020 to 9.3% (*n* = 201), *χ*^2^ = 9.198, *df* = 1, *p* < 0.01. Prevalence of regular smoking decreased from 1.7% (*n* = 37) to 0.8% (*n* = 17), *χ*^2^ = 7.905 *df* = 1, *p* < 0.01. Smoking susceptibility increased from 18.0% (*n* = 336) in 2020 to 21.5% (*n* = 421), *χ*^2^ = 7.093, *df* = 1, *p* < 0.01.

#### 3.1.2 Vaping

Prevalence of having ever tried vaping increased from 10.1% (*n* = 215) in 2020 to 17.5% (*n* = 378), *χ*^2^ = 48.278, *df* = 1, *p* < 0.001 and regular vaping (at least weekly) increased from 1.0% (*n* = 22) in 2020 to 2.6% (*n* = 57) in 2023, *χ*^2^ = 15.092, *df* = 1, *p* < 0.001. Vaping susceptibility increased from 33.0% (*n* = 629) in 2020 to 43.2% (*n* = 771), *χ*^2^ = 40.495, *df* = 1, *p* < 0.001.

#### 3.1.3 Vaping and/or smoking status

Most young people had never vaped nor smoked, though this decreased from 84.5% (*n* = 1792) in 2020 to 80.8% (*n* = 1748) in 2023, *χ*^2^ = 10.077, *df* = 1, *p* = 0.002. Prevalence of having vaped but never smoked increased from 3.3% (*n* = 71) in 2020 to 9.9% (*n* = 214) in 2023, *χ*^2^ = 73.897, *df* = 1, *p* < 0.001.

### 3.2 Associations between smoking and vaping

In both 2020 and 2023 there was an association between having tried vaping and being susceptible to smoking, with more than 40% of ever vapers being susceptible to smoking. In 2020, 40.8% (*n* = 29) of young ever vapers were categorised susceptible to smoke compared with 17.1% (*n* = 307) amongst young people who have never vaped *χ*^2^ = 25.979, *df* = 1, *p* < 0.001. In 2023, 43.0% (*n* = 92) of young ever vapers were categorised susceptible to smoke compared with 18.9% (*n* = 330) amongst young never vapers *χ*^2^ = 65.658, *df* = 1, *p* < 0.001.

In 2020, 3.8% of never smokers (*n* = 71) had tried vaping and 55.8% of ever smokers (*n* = 144) had tried vaping, *χ*^2^ = 672.770, *df* = 1, *p* < 0.001. By 2023, 10.9% of never smokers (*n* = 214) and 81.6% of ever smokers (*n* = 164) had tried vaping, *χ*^2^ = 631.646, *df* = 1, *p* < 0.001. Vaping experimentation was not confined to young people who had already tried smoking: in 2020, never smokers accounted for a third of ever vapers (*n* = 71, 33.0%), by 2023 never smokers accounted for the majority of ever vapers (*n* = 214, 56.6%).

### 3.3 Vaping devices used when first vaped

Amongst ever vapers in 2023 (*n* = 378, weighted), the majority 72.8% (*n* = 275) indicated having used a disposable the first time. Rechargeable pod style devices were used by 11.7% (*n* = 44) on the first occasion whilst 6.8% (*n* = 26) used a rechargeable tank device (Table 1). Around one in 10 (9.6%, *n* = 33) were unable to indicate the type of device they had first used.

### 3.4 Vaping devices used in the past 4 weeks

Amongst young people who had vaped in the past month (*n* = 192, weighted), the most popular type of vape used was a disposable with the majority (76.5%, *n* = 147) having used disposables. Rechargeable pod style devices had been used by around a fifth (21.7%, *n* = 42) whilst around one in eight had used a tank device (12.4%, *n* = 24). A small proportion, 5.1% (*n* = 10), were unable to indicate the type of device(s) they used in the past 4 weeks.

### 3.5 Rating of devices/products

Participants indicated some uncertainty around appeal of products to never smokers and nicotine content of and environmental harms from tanks and disposables. Between 9 and 20% answered ‘not sure’ rather than providing a rating (Supplementary Table S2).

Over half the young people perceived disposables to be appealing to (*n* = 1,091, 53.0%) and popular with their age group (*n* = 1,098, 54.6%) (Table 2) and over two-fifths perceived disposables as appealing to never smokers (*n* = 866, 45.8%).

**Table 2:** Proportion of young people giving favourable device/product ratings (scores 4 and 5).

	Tank		Disposable		Cigarette	
	*n*	% [95% CI]	*n*	% [95% CI]	*n*	% [95% CI]
% Scoring 4 or 5 and rating as:						
Appeal						
(a) Would appeal to people my age	(596)	29.3% [27.3, 31.3]	(1,091)	53.0% [50.9, 55.2]	(383)	18.5% [16.8, 20.2]
(b) Popular with people my age	(483)	24.3% [22.4, 26.2]	(1,098)	54.6% [52.5, 56.8]	(230)	11.3% [9.9, 12.7]
(c) Would appeal to people who have never smoked	(501)	26.9% [24.9, 28.9]	(866)	45.8% [43.5, 48.0]	(361)	18.9% [17.1, 20.7]
(d) I would be tempted to use this	(216)	10.3% [9.0, 11.6]	(354)	16.9% [15.3, 18.5]	(144)	6.8% [5.7, 7.9]
Image						
(e) Cool	(260)	12.8% [11.4, 14.3]	(425)	20.6% [18.9, 22.4]	(78)	3.7% [2.9, 4.5]
(f) Fun	(191)	9.4% [8.2, 10.7]	(388)	19.0% [17.3, 20.7]	(53)	2.6% [1.9, 3.3]
(g) Looks pleasant to use	(220)	10.7% [9.4, 12.0]	(556)	26.9% [25.0, 28.8]	(56)	2.6% [2.0, 3.3]
Harms						
(h) Not at all harmful to health	(187)	9.3% [8.0, 10.5]	(253)	12.5% [11.1, 13.9]	(22)	1.0% [0.6, 1.4]
(i) Not at all harmful to the environment	(293)	15.0% [13.4, 16.6]	(256)	13.1% [11.6, 14.6]	(131)	6.5% [5.4, 7.5]
(j) Not at all addictive	(182)	9.2% [7.9, 10.5]	(191)	9.6% [8.3, 10.8]	(42)	2.0% [1.4, 2.6]
(k) Unlikely to contain nicotine	(130)	7.5% [6.3, 8.7]	(211)	11.7% [10.2, 13.2]	(57)	2.7% [2.0, 3.4]

Base: All YEPS 2023 participants (*n* = 2,164, weighted), with valid responses. Missing values range from 44 to 428 due to ‘not sure’ responses.

Around a fifth or more held a positive image of disposables, considering them cool (*n* = 425, 20.6%), fun (*n* = 388, 19.0%) and looking pleasant to use (*n* = 556, 26.9%).

Around a tenth or more perceived disposables as not being harmful, including being not at all addictive (*n* = 191, 9.6%,) and not harmful to the environment (*n* = 256, 13.1%).

The mean scores (Table 3) indicate, average ratings of all devices being at the unfavourable end of the scales, except for the appeal scores for disposables which rated at the favourable end. The paired analyses (Table 3) and the GEE analyses (Supplementary Tables S3a–S3c) show that disposables are consistently rated more favourably than tank devices (range Adjusted Odds Ratio (AOR): 1.40 to 4.16; *p* < 0.001) except for addictiveness where they did not differ and harm to the environment where disposables rated less favourably than the tank (AOR = 0.83; *p* < 0.001). Cigarettes were consistently rated less favourably than the tank (range AOR: 0.08 to 0.61; *p* < 0.001).

**Table 3:** Mean ratings on response to ‘Disposable’ vape versus ‘Tank’ device and ‘Tank’ device versus traditional ‘Cigarette’.

	Disposable vs. Tank					Tank vs. Cigarette				
	*n*	Disposable Mean [95% CI] SD	Tank Mean [95% CI] SD	Effect Size (*r*)^#^	*p* value*	*n*	Tank Mean [95% CI] SD	Cigarette Mean [95% CI] SD	Effect Size (*r*)^#^	*p* value*
(a) Would not appeal to people my age (1)/Would appeal to people my age (5)	2,024	3.39 [3.32, 3.45] *1.53*	2.59 [2.53, 2.66] *1.48*	0.41	<0.001	2,010	2.59 [2.53, 2.66] *1.48*	2.11 [2.05, 2.17] *1.39*	0.28	<0.001
(b) Unpopular with people my age (1)/Popular with people my age (5)	1,976	3.47 [3.40, 3.53] *1.47*	2.51 [2.45, 2.57] *1.42*	0.55	<0.001	1,966	2.51 [2.44, 2.57] *1.42*	1.88 [1.82, 1.93] *1.21*	0.40	<0.001
(c) Would not appeal to people who have never smoked (1)/Would appeal to people who have never smoked (5)	1,835	3.15 [3.08, 3.22] *1.51*	2.55 [2.48, 2.62] *1.47*	0.40	<0.001	1,796	2.55 [2.49, 2.62] *1.47*	2.13 [2.06, 2.19] *1.44*	0.26	<0.001
(d) I would not be tempted to use this (1)/I would be tempted to use this (5)	2,090	1.91 [1.85, 1.97] *1.37*	1.62 [1.57, 1.67] *1.17*	0.25	<0.001	2,090	1.62 [1.57, 1.67] *1.17*	1.37 [1.33, 1.42] *0.99*	0.22	<0.001
(e) Not cool (1)/Cool (5)	2,013	2.15 [2.09, 2.21] *1.42*	1.87 [1.81, 1.92] *1.27*	0.24	<0.001	2,002	1.86 [1.80, 1.91] *1.27*	1.32 [1.28, 1.35] *0.83*	0.38	<0.001
(f) Boring (1)/Fun (5)	2,010	2.15 [2.09, 2.21] *1.37*	1.83 [1.78, 1.88] *1.14*	0.32	<0.001	1,985	1.81 [1.76, 1.86] *1.14*	1.35 [1.32, 1.38] *0.78*	0.40	<0.001
(g) Looks unpleasant to use (1)/Looks pleasant to use (5)	2,042	2.44 [2.38, 2.51] *1.50*	1.79 [1.74, 1.84] *1.19*	0.46	<0.001	2,050	1.78 [1.73, 1.83] *1.18*	1.28 [1.25, 1.31] *0.74*	0.38	<0.001
(h) Very harmful to health (1)/Not at all harmful to health (5)	2,002	2.11 [2.06, 2.16] *1.15*	1.97 [1.92, 2.01] *1.09*	0.20	<0.001	2,009	1.96 [1.92, 2.01] *1.08*	1.13 [1.11, 1.15] *0.49*	0.60	<0.001
(i) Very harmful to the environment (1)/Not at all harmful to the environment (5)	1,935	2.05 [1.99, 2.10] *1.22*	2.13 [2.08, 2.19] *1.25*	0.13	<0.001	1,908	2.12 [2.07, 2.18] *1.25*	1.62 [1.58, 1.66] *0.99*	0.36	<0.001
(j) Very addictive (1)/Not at all addictive (5)	1,972	1.88 [1.83, 1.93] *1.13*	1.89 [1.84, 1.94] *1.13*	0.01	Ns	1,963	1.89 [1.84, 1.94] *1.12*	1.22 [1.19, 1.25] *0.66*	0.54	<0.001
(k) Likely to contain nicotine (1)/Unlikely to contain nicotine (5)	1,714	1.75 [1.69, 1.80] *1.16*	1.56 [1.52, 1.61] *1.02*	0.21	<0.001	1,722	1.56 [1.52, 1.61] *1.02*	1.17 [1.14, 1.20] *0.67*	0.38	<0.001

Paired means are from weighted data. *Wilcoxon signed rank test for significant difference, conducted on unweighted data. ^#^Effect size estimate *r*, for Wilcoxon signed rank test, *r* = *Z*/√*N*.

## 4 Discussion

Between 2020 and 2023, young people’s smoking prevalence decreased, whilst vaping prevalence increased, supporting trends found in other studies (1, 2). Vaping and smoking susceptibility increased and there was an association between ever tried vaping and susceptibility to smoke. Across 11 items exploring product appeal, imagery and perceptions of harm, young people rated a cigarette less favourably than a tank model. They rated a disposable device more favourably than a tank model, on all but two of the items (perceptions of addictiveness and harm to the environment). Positive ratings of the disposable device were most pronounced on items indicating youth appeal, i.e., ‘popular with people my age’, ‘would appeal to people my age’ and ‘would appeal to people who have never smoked’. Prior to the emergence of brands such as Elf Bar on the market, surveys consistently found limited youth vaping amongst never smokers (21). Our study shows that young people’s more positive perceptions of disposable vapes, relative to their perceptions of an earlier generation of vaping product (tank model), may have contributed to observed increases in youth vaping. Young people indicated uncertainty around the nicotine content in vapes. This is understandable given that different strengths of e-liquid can be placed in tanks, and non-nicotine versions of disposables are available. However, this uncertainty may reflect young people’s reality should they be offered a puff.

To circumvent the disposable vapes ban, many vape companies traditionally focusing on disposables have launched rechargeable pod models to appeal to their existing customer base (e.g., Lost Mary Tappo and Elf Bar Elfa Pro) (22). Almost identical in appearance, and priced similarly to, their disposable counterparts, these products may hold similar youth appeal. As vaping has become an established norm amongst some young people, and with brand loyalty to the most prominent brands (9), young people’s product engagement could simply shift from disposables to a rechargeable version of the same brand. The disposables ban is unlikely to be a silver bullet to tackle youth vaping. Like tobacco, a comprehensive policy approach is needed to further restrict vape advertising and promotion. If the existing regulatory framework needs to be strengthened to protect young people, it may be challenging for policymakers to maintain the UK’s balanced approach to regulation and ensure that vapes are also accessible to adults who may benefit from switching. However, there is emerging evidence that some policy measures may not necessarily conflict and may offer a ‘win-win’ solution. For example, adults in England and Scotland who vaped and/or smoked believed that the number and/or types of shops selling vapes could be reduced without affecting accessibility to vapes (8). Many also believed that positioning vapes behind the counter would not be problematic for them, whilst communicating an age-restricted product for young people. Additionally, some adults who vaped were supportive of a vape display ban (8). Standardised (plain) packaging has the potential to reduce interest in trying vapes amongst young people, without reducing their appeal amongst adults (23, 24). Evaluation of the impact of the disposables ban on youth nicotine and tobacco use will be critical. It will also be important to monitor market developments so public health can be alert to emerging issues. The rapid growth in the disposables market highlights how quickly products can take off. Although use of heated tobacco in the UK is rare (25), new products are entering the market, alongside increased sales, and investment from companies (26, 27). Similar is evidenced for nicotine pouches (28), for which youth awareness and use amongst young adults is increasing (29). It will be important to monitor youth response to these and any future emerging nicotine products.

Given the cross-sectional design of the study, the study does not demonstrate causality in the association between vaping and smoking susceptibility. This association was found in 2020 and 2023, however, with larger numbers of young people vaping since 2023, this may now be more of a concern. Whilst susceptibility cannot tell us that young never-smokers will definitely go on to smoke, it is a well-validated measure of smoking intent (18). Longitudinal research is needed to investigate the direction of this association and possible mediating factors. Nevertheless, this study provides evidence of population-level changes in susceptibility across the UK. With the current policy focus on youth vaping and given that susceptibility to both vaping and smoking increased in our study timeframe, comprehensive monitoring of youth tobacco use, as well as vaping, is still critical, especially given the recent rise in 11–17-year-olds reporting having tried smoking (1). Renewed public health messaging which emphasises the factors influencing youth vaping susceptibility may also galvanise collaboration amongst stakeholders for more effective prevention strategies.

The study has other limitations. The surveys use non-probability samples, which have issues for generalisability and prevalence estimates (30). Whilst we cannot say that the sample is completely representative of young people in the UK, the sample is large enough to be indicative of the UK adolescent population, and sex, age and smoking prevalence were broadly comparable with national data available from 2021 (31). The study relies on self-reported data which could introduce bias. However, we sought to minimise this through careful survey design and by reassuring participants that they would not be identified. Recruitment and online survey administration method may have resulted in social desirability bias. Recruitment of 11–15-year-olds was via their parent on the YouGov panel. Surveys may have been completed at home, with a family member present. This may have influenced participants’ perceptions about tobacco and vapes. However, the survey is designed to be completed on any device. Completing on a mobile phone may have helped protect participants’ privacy. The findings are also limited to the three products shown in the survey. A tank model was chosen to reflect an earlier generation product and one arguably more associated with smoking cessation (the primary purpose of vaping), but there may have been different results should we have explored ratings of a refillable cartridge or pod device. Going forward, it will be crucial to monitor youth perceptions of refillable cartridge and pod devices given their prominence on the market as replacements since implementation of the disposable vapes ban.

## 5 Conclusion

Approximately 2 years after the introduction of disposable vapes to the UK, there was an increase in young people’s vaping experimentation, and young people held more favourable views of disposable vapes relative to tank devices, and cigarettes, across most measures of appeal, imagery and harm. Further monitoring of the nicotine market, product marketing, and young people’s response is critical, particularly as the market adapts to the UK disposable vapes ban, and implementation of some of the Tobacco and Vapes Bill measures to deter youth vaping may take some time.

## Data Availability

The dataset used and analysed is available from the corresponding author on reasonable request.
