# Supplementary material for: Young people’s smoking and vaping behaviour, and comparative perceptions of appeal, imagery and harm, across different vape devices and a tobacco cigarette: findings from UK cross-sectional surveys in 2020 and 2023
Source: Front Public Health. 2025 Dec 18;13:1689766. doi: 10.3389/fpubh.2025.1689766 (PMC12756415; doi:10.3389/fpubh.2025.1689766)
Supplement: Supplementary file 1 [file Data_Sheet_1.pdf]

Supplementary Material

Supplementary Table S1: Survey response overview

	YTPS 2020	YEPS 2023
**Opened the survey invitation**	**3,290**	**3822**
Screened out	323	454
**Eligible respondents who commenced survey**	**2,967**	**3,368**
Dropped out during survey	820	1,118
**Number of participants who completed survey**	**2,147**	**2,250**
Total participants removed through cleaning	26	86
**Final number of completed responses**	**2,121**	**2,164**

Supplementary Table S2: Proportion of young people answering 'not sure' on device/product ratings

		Tank		Disposable		Cigarette	
		(n)	%	(n)	%	(n)	%
Appeal							
a)	Would not appeal to people my age (1) / Would appeal to people my age (5)	(132)	6%	(106)	5%	(95)	4%
b)	Unpopular with people my age (1) / Popular with people my age (5)	(179)	8%	(155)	7%	(126)	6%
c)	Would not appeal to people who have never smoked (1) / Would appeal to people who have never smoked (5)	(304)	14%	(272)	13%	(252)	12%
d)	I would not be tempted to use this (1) / I would be tempted to use this (5)	(65)	3%	(65)	3%	(47)	2%
Image							
e)	Not cool (1) / Cool (5)	(140)	6.5%	(107)	5%	(62)	3%
f)	Boring (1) / Fun (5)	(146)	7%	(125)	6%	(121)	6%
g)	Looks unpleasant to use (1) / Looks pleasant to use (5)	(103)	5%	(98)	5%	(53)	2%
Harms							
h)	Very harmful to health (1) / Not at all harmful to health (5)	(147)	7%	(139)	6%	(44)	2%
i)	Very harmful to the environment (1) / Not at all harmful to the environment (5)	(214)	10%	(204)	9%	(139)	6%
j)	Very addictive (1) / Not at all addictive (5)	(182)	8%	(163)	8%	(71)	3%
k)	Likely to contain nicotine (1) / Unlikely to contain nicotine (5)	(428)	20%	(352)	16%	(89)	4%

Base: all YEPS 2023 (n=2164, weighted)

Supplementary Table S3a: General estimating equations for binary outcomes: perceptions of disposable vape and cigarette versus tank vaping device: Appeal

	a<br>Appeal to their age<br>1= Would appeal to people my age (score 4-5)<br>0= Neutral or would not (score ≤3)				b<br>Popularity<br>1= Popular with my age (score 4-5)<br>0= Neutral or not (score ≤3)			
	N	AOR*	95% CI	P	N	AOR*	95% CI	P
**Device/product**								
Cigarette	2069	0.52	[0.45-0.59]	<0.001	2042	0.34	[0.29-0.41]	<0.001
Disposable	2059	2.84	[2.54-3.17]	<0.001	2015	4.16	[3.72-4.64]	<0.001
Tank	2032	Ref			1989	ref		
**Sex**								
Female	3054	1.11	[0.98-1.27]	0.105	3027	1.10	[0.94-1.28]	0.220
Male	3106	Ref			3019	Ref		
**Age group**								
15-16	1942	1.59	[1.34-1.88]	<0.001	1930	2.44	[1.99-2.99]	<0.001
13-14	2076	1.53	[1.30-1.81]	<0.001	2029	2.40	[1.97-2.93]	<0.001
11-12	2142	Ref			2087	ref		
**Social Grade**								
C2DE	1551	1.08	[0.93-1.27]	0.310	1497	1.18	[0.99-1.40]	0.064
ABC1	4609	Ref			4549	Ref		
**Vaping and/or smoking status**								
Ever smoked but never vaped	101	2.03	[1.19-3.45]	0.009	99	1.26	[0.65-2.46]	0.492
Ever vaped and ever smoked	435	1.44	[1.14-1.81]	0.002	439	2.54	[1.97-3.28]	<0.001
Ever vaped but never smoked	606	1.43	[1.18-1.72]	<0.001	604	2.11	[1.71-2.59]	<0.001
Never smoked nor vaped	5018	Ref			4904	Ref		

* adjusted for all other variables in the model, AOR, adjusted odds ratio; ref, reference category; 95% CI, 95% confidence interval: Valid cases a) n=6160, missing=332; b) n=6046, missing=446. Data are unweighted as key demographics are controlled for in the analysis.

Supplementary Table S3a Cont'd: General estimating equations for binary outcomes: perceptions of disposable vape and cigarette versus tank vaping device: Appeal

	c<br>Appeal to never smokers<br>1= Would appeal to never smokers (score 4-5)<br>0= Neutral or would not (score ≤3)				d<br>Temptation<br>1= Would be tempted to use (score 4-5)<br>0= Neutral or would not (score ≤3)			
	**N**	**AOR***	**95% CI**	**P**	**N**	**AOR***	**95% CI**	**P**
**Device/product**								
Cigarette	1910	0.61	[0.54-0.70]	<0.001	2120	0.57	[0.47-0.71]	<0.001
Disposable	1888	2.32	[2.10-2.57]	<0.001	2103	1.94	[1.65-2.28]	<0.001
Tank	1857	Ref			2101	ref		
**Sex**								
Female	2789	1.00	[0.86-1.17]	0.962	3125	0.88	[0.72-1.09]	0.257
Male	2866	Ref			3199	Ref		
**Age group**								
15-16	1858	1.20	[0.99-1.45]	0.060	1999	0.95	[0.72-1.26]	0.736
13-14	1911	1.31	[1.08-1.58]	0.005	2129	0.95	[0.72-1.26]	0.742
11-12	1886	Ref			2196	ref		
**Social Grade**								
C2DE	1396	1.25	[1.06-1.49]	0.010	1596	1.09	[0.87-1.38]	0.448
ABC1	4259	Ref			4728	Ref		
**Vaping and/or smoking status**								
Ever smoked but never vaped	94	1.43	[0.77-2.64]	0.252	97	2.62	[1.17-5.84]	0.019
Ever vaped and ever smoked	417	1.47	[1.13-1.91]	0.004	446	12.42	[9.32-16.54]	<0.001
Ever vaped but never smoked	569	1.43	[1.15-1.77]	0.001	616	5.89	[4.49-7.72]	<0.001
Never smoked nor vaped	4575	Ref			5165	Ref		

* adjusted for all other variables in the model, AOR, adjusted odds ratio; ref, reference category; 95% CI, 95% confidence interval: Valid cases c) n=5655, missing=837; d) n=6324, missing=168. . Data are unweighted as key demographics are controlled for in the analysis.

Supplementary Table S3b: General estimating equations for binary outcomes: perceptions of disposable vape and cigarette versus tank vaping device: Image

	e				f			
	Cool				Fun			
	1= Cool (score 4-5)				1= Fun (score 4-5)			
	0= Neutral or not cool (score ≤3)				0= Neutral or boring (score ≤3)			
	**N**	**AOR***	**95% CI**	**P**	**N**	**AOR***	**95% CI**	**P**
**Device/product**								
Cigarette	2106	0.23	[0.18-0.31]	<0.001	2048	0.25	[0.18-0.34]	<0.001
Disposable	2059	1.99	[1.69-2.34]	<0.001	2043	2.70	[2.29-3.18]	<0.001
Tank	2023	Ref			2023	ref		
**Sex**								
Female	3045	0.93	[0.77-1.13]	0.489	3018	0.97	[0.77-1.21]	0.769
Male	3143	Ref			3096	Ref		
**Age group**								
15-16	1968	0.55	[0.42-0.71]	<0.001	1948	0.73	[0.55-0.98]	0.038
13-14	2096	0.81	[0.64-1.02]	0.078	2037	0.75	[0.56-0.99]	0.044
11-12	2124	Ref			2129	ref		
**Social Grade**								
C2DE	1559	1.01	[0.81-1.26]	0.924	1537	0.97	[0.75-1.24]	0.786
ABC1	4629	Ref			4577	Ref		
**Vaping and/or smoking status**								
Ever smoked but never vaped	95	5.68	[3.04-10.61]	<0.001	93	2.70	[1.15-6.37]	0.023
Ever vaped and ever smoked	441	11.63	[8.76-15.45]	<0.001	433	14.56	[10.47-20.25]	<0.001
Ever vaped but never smoked	608	4.25	[3.28-5.51]	<0.001	594	7.19	[5.32-9.71]	<0.001
Never smoked nor vaped	5044	Ref			4994	Ref		

* adjusted for all other variables in the model, AOR, adjusted odds ratio; ref, reference category; 95% CI, 95% confidence interval: Valid cases e) n=6188, missing=304; f) n=6114, missing=378. Data are unweighted as key demographics are controlled for in the analysis.

Supplementary Table S3b Cont'd: General estimating equations for binary outcomes: perceptions of disposable vape and cigarette versus tank vaping device: Image

	g			
	Pleasant<br>1= Looks pleasant to use (score 4-5)<br>0= Neutral or unpleasant (score ≤3)			
	N	AOR*	95% CI	P
**Device/product**				
Cigarette	2112	0.21	[0.16-0.29]	<0.001
Disposable	2066	3.81	[3.22-4.49]	<0.001
Tank	2062	Ref		
**Sex**				
Female	3088	0.96	[0.79-1.16]	0.651
Male	3152	Ref		
**Age group**				
15-16	1967	0.68	[0.53-0.87]	0.002
13-14	2108	0.79	[0.63-1.01]	0.055
11-12	2165	Ref		
**Social Grade**				
C2DE	1572	1.09	[0.88-1.35]	0.445
ABC1	4668	Ref		
**Vaping and/or smoking status**				
Ever smoked but never vaped	94	3.34	[1.62-6.89]	0.001
Ever vaped and ever smoked	440	11.69	[8.55-15.97]	<0.001
Ever vaped but never smoked	608	5.31	[4.11-6.86]	<0.001
Never smoked nor vaped	5098	Ref		

* adjusted for all other variables in the model, AOR, adjusted odds ratio; ref, reference category; 95% CI, 95% confidence interval:

Valid cases g) n=6240, missing=252. Data are unweighted as key demographics are controlled for in the analysis.

Supplementary Table S3c: General estimating equations for binary outcomes: perceptions of disposable vape and cigarette versus tank vaping device: Harms

	h<br>Harm to health<br>1= Not harmful to health (score 4-5)<br>0= Neutral or harmful (score ≤3)				i<br>Harm to environment<br>1= Not harmful to environment (score 4-5)<br>0= Neutral or harmful (score ≤3)			
	**N**	**AOR***	**95% CI**	**P**	**N**	**AOR***	**95% CI**	**P**
**Device/product**								
Cigarette	2123	0.08	[0.05-0.13]	<0.001	2028	0.38	[0.31-0.46]	<0.001
Disposable	2027	1.40	[1.22-1.61]	<0.001	1959	0.83	[0.75-0.92]	<0.001
Tank	2021	Ref			1948	ref		
**Sex**								
Female	3064	0.86	[0.66-1.13]	0.278	2941	0.77	[0.61-0.97]	0.029
Male	3107	Ref			2994	Ref		
**Age group**								
15-16	1960	0.60	[0.43-0.85]	0.004	1905	0.69	[0.51-0.94]	0.018
13-14	2091	0.53	[0.38-0.75]	<0.001	1998	0.91	[0.69-1.22]	0.543
11-12	2120	Ref			2032	ref		
**Social Grade**								
C2DE	1555	0.88	[0.64-1.21]	0.432	1484	1.00	[0.76-1.31]	0.992
ABC1	4616	Ref			4451	Ref		
**Vaping and/or smoking status**								
Ever smoked but never vaped	95	2.08	[0.76-5.69]	0.156	98	1.45	[0.58-3.60]	0.423
Ever vaped and ever smoked	436	7.77	[5.33-11.33]	<0.001	413	4.93	[3.50-6.92]	<0.001
Ever vaped but never smoked	611	5.34	[3.73-7.64]	<0.001	579	3.03	[2.16-4.26]	<0.001
Never smoked nor vaped	5029	Ref			4845	Ref		

* adjusted for all other variables in the model, AOR, adjusted odds ratio; ref, reference category; 95% CI, 95% confidence interval: Valid cases cases h) n=6171, missing=321; i) n=5935, missing=557. Data are unweighted as key demographics are controlled for in the analysis.

Supplementary Table S3c Cont'd: General estimating equations for binary outcomes: perceptions of disposable vape and cigarette versus tank vaping device: Harms

	j Addictiveness 1= Not addictive (score 4-5) 0= Neutral or addictive (score ≤3)				k Likelihood contains nicotine 1= Unlikely to contain nicotine (score 4-5) 0= Neutral or likely to contain (score ≤3)			
	**N**	**AOR***	**95% CI**	**P**	**N**	**AOR***	**95% CI**	**P**
**Device/product**								
Cigarette	2097	0.22	[0.16-0.29]	<0.001	2077	0.30	[0.22-0.41]	<0.001
Disposable	2008	1.03	[0.89-1.19]	0.676	1802	1.70	[1.43-2.02]	<0.001
Tank	1989	Ref			1724	ref		
**Sex**								
Female	3020	0.70	[0.53-0.93]	0.015	2777	0.91	[0.70-1.19]	0.509
Male	3074	Ref			2826	Ref		
**Age group**								
15-16	1949	0.61	[0.41-0.88]	0.009	1798	0.57	[0.40-0.80]	0.001
13-14	2063	0.54	[0.38-0.76]	0.001	1918	0.79	[0.58-1.09]	0.149
11-12	2082	Ref			1887	ref		
**Social Grade**								
C2DE	1531	0.98	[0.71-1.37]	0.926	1423	0.88	[0.64-1.21]	0.439
ABC1	4563	Ref			4180	Ref		
**Vaping and/or smoking status**								
Ever smoked but never vaped	95	2.44	[0.88-6.74]	0.086	89	0.80	[0.30-2.13]	0.654
Ever vaped and ever smoked	436	4.08	[2.64-6.30]	<0.001	424	1.17	[0.71-1.91]	0.535
Ever vaped but never smoked	597	2.65	[1.73-4.04]	<0.001	582	1.60	[1.08-2.36]	0.018
Never smoked nor vaped	4966	Ref			4508	Ref		

* adjusted for all other variables in the model, AOR, adjusted odds ratio; ref, reference category; 95% CI, 95% confidence interval: Valid cases j) n=6094, missing=398; k) n=5603, missing=889. Data are unweighted as key demographics are controlled for in the analysis.
